# Sexual Orientation Disparities in Risk Factors for Adverse COVID-19–Related Outcomes, by Race/Ethnicity — Behavioral Risk Factor Surveillance System, United States, 2017–2019

**DOI:** 10.15585/mmwr.mm7005a1

**Published:** 2021-02-05

**Authors:** Kevin C. Heslin, Jeffrey E. Hall

**Affiliations:** 1 CDC COVID-19 Response Team.

Sexual minority persons experience health disparities associated with sexual stigma and discrimination and have a high prevalence of several health conditions that have been associated with severe coronavirus disease 2019 (COVID-19) (*1*,*2*). Current COVID-19 surveillance systems do not capture information about sexual orientation. To begin bridging the gap in knowledge about COVID-19 risk among sexual minority adults, CDC examined disparities between sexual minority and heterosexual adults in the prevalence of underlying conditions with strong or mixed evidence of associations with severe COVID-19–related illness (*3*), by using data from the 2017–2019 Behavioral Risk Factor Surveillance System (BRFSS).* When age, sex, and survey year are adjusted, sexual minority persons have higher prevalences than do heterosexual persons of self-reported cancer, kidney disease, chronic obstructive pulmonary disease (COPD), heart disease (including myocardial infarction, angina, or coronary heart disease), obesity, smoking, diabetes, asthma, hypertension, and stroke. Sexual minority adults who are members of racial/ethnic minority groups disproportionately affected by the pandemic also have higher prevalences of several of these health conditions than do racial/ethnic minority adults who are heterosexual. Collecting data on sexual orientation in COVID-19 surveillance and other studies would improve knowledge about disparities in infection and adverse outcomes by sexual orientation, thereby informing more equitable responses to the pandemic.

Conducted by the 50 states, the District of Columbia, three U.S. territories, and two freely associated states, BRFSS is a collection of population health surveys that gather demographic and health-related information from noninstitutionalized U.S. residents aged ≥18 years. BRFSS includes standard core questions and optional modules. All participants are asked “Has a doctor, nurse, or other health practitioner ever told you that you have…” followed by a list of health conditions.^†^ The number of jurisdictions opting to include questions on sexual orientation in BRFSS has increased in recent years. Gender identity is addressed in a BRFSS survey question separately from sexual orientation questions. This analysis combined the 3 most recent years of BRFSS data for states that include a sexual orientation question: a total of 28 states in 2017, a total of 29 states in 2018, and a total of 31 states in 2019.**^§^** The percentage of BRFSS respondents who refused to answer the sexual orientation question was 1.8% (both male and female) in 2017, 1.5% (male) and 1.9% (female) in 2018, and 1.6% (male) and 2.0% (female) in 2019. Among states with a sexual orientation question, the median overall survey response rate was 42.3% in 2017, 48.5% in 2018, and 46.4% in 2019.

For this analysis, respondents were classified as sexual minority persons (versus heterosexual persons) if they selected any of the following responses from the 2017–2019 questions on sexual orientation: “gay,” “lesbian or gay,” or “bisexual^¶^” (sexual minority: 24,582 [unweighted], 4.7% [weighted]; heterosexual: 619,374 [unweighted], 95.3% [weighted]). Race and ethnicity were categorized as Hispanic (any race), non-Hispanic Black, non-Hispanic White, and non-Hispanic other; the non-Hispanic other category includes non-Hispanic Asian, non-Hispanic American Indian/Alaskan Native, and non-Hispanic persons of other races/ethnicities. Adults with the following conditions are at increased risk for severe illness from COVID-19: cancer, chronic kidney disease, COPD, heart conditions, obesity, pregnancy, sickle cell disease, smoking, and type 2 diabetes mellitus (*3*). In addition, adults with the following conditions might be at increased risk for severe illness from COVID-19: asthma, cerebrovascular disease, cystic fibrosis, hypertension, immunocompromised state, neurologic conditions (e.g., dementia), liver disease, overweight, pulmonary fibrosis, thalassemia, and type 1 diabetes mellitus. Among these conditions with strong or mixed evidence of associations with adverse COVID-19–related outcomes (*3*,*4*), the following variables from the BRFSS core module were included: asthma (current and ever), cancer (except nonmelanoma skin cancer), COPD, heart disease (myocardial infarction, angina, or coronary heart disease) (*4*), diabetes, hypertension, kidney disease, obesity (current), smoking (current), and stroke. Hypertension questions were asked only in 2017 and 2019.**

Adjusted percentages and adjusted prevalence ratios (aPRs) comparing sexual minority persons and heterosexual persons with each condition were calculated overall and stratified by race/ethnicity. Using Stata (version 16.0; StataCorp) software to account for the BRFSS survey design, all estimates were adjusted for age, sex (male or female), and survey year, using multivariate logistic regression with the margins and nonlinear combination of estimators (nlcom) postestimation commands. The nlcom procedure takes nonlinear transformations of a parameter estimate from a fitted model and applies the delta method to calculate the variance. All aPRs with 95% confidence intervals that exclude 1 are considered statistically significant.

Among all racial/ethnic groups combined, sexual minority persons had higher adjusted prevalences of asthma (current and ever), cancer, heart disease, COPD, hypertension, kidney disease, obesity (current), smoking (current), and stroke than did heterosexual persons (Table). Among non-Hispanic Black persons, sexual minority persons had higher adjusted prevalences of asthma (current and ever), COPD, and smoking (current) than did heterosexual persons. Among non-Hispanic White persons, sexual minority persons had higher adjusted prevalences of asthma (current and ever), cancer, COPD, diabetes, hypertension, kidney disease, obesity (current), smoking (current), and stroke than did heterosexual persons. Among Hispanic persons, sexual minority persons had higher adjusted prevalences of asthma (current and ever), cancer, COPD, smoking (current), and stroke than did heterosexual persons. Among non-Hispanic other persons, sexual minority persons had higher adjusted prevalences of asthma (current and ever), cancer, heart disease, COPD, obesity (current), and smoking (current) than did heterosexual persons. Among the 11 conditions studied, the highest significant aPRs were observed among sexual minority persons overall, and for eight of these 11 conditions, the highest significant aPRs were among sexual minority persons within a racial/ethnic minority group. None of the 11 conditions studied was more prevalent among heterosexual persons than among members of sexual minority groups.

**TABLE:** Adjusted prevalence and adjusted prevalence ratios (aPRs)* of underlying health conditions^†^ among sexual minority^§^ and heterosexual adults, by race and Hispanic origin — Behavioral Risk Factor Surveillance System, United States, 2017–2019

Characteristic	% (95% CI)				
	All	Black, non-Hispanic	White, non-Hispanic	Other, non-Hispanic	Hispanic
Respondents, no.	643,956	54,486	495,278	51,781	42,411
Sexual minority persons,^¶^ no. (%)	24,582 (4.7)	2,004 (4.7)	17,656 (4.4)	2,616 (5.5)	2,306 (5.3)
**Underlying condition**					
**Asthma, current**					
Sexual minority	13.8 (13.0–14.6)	14.5 (12.2–16.8)	13.3 (12.4–14.3)	13.5 (11.1–16.0)	14.2 (11.5–16.9)
Heterosexual	8.9 (8.8–9.1)	10.7 (10.2–11.2)	9.2 (9.0–9.3)	8.1 (7.5–8.7)	6.8 (6.3–7.3)
aPR	1.55 (1.45–1.64)	1.35 (1.13–1.58)	1.46 (1.35–1.56)	1.67 (1.35–2.00)	2.09 (1.67–2.51)
**Asthma, ever**					
Sexual minority	19.8 (18.8–20.8)	21.0 (18.2–23.8)	19.1 (18.0–20.1)	19.7 (16.7–22.8)	20.9 (17.7–24.0)
Heterosexual	14.1 (13.9–14.2)	15.9 (15.3–16.5)	14.3 (14.1–14.6)	13.3 (12.5–14.0)	11.8 (11.2–12.4)
aPR	1.41 (1.34–1.48)	1.32 (1.14–1.50)	1.33 (1.25–1.41)	1.49 (1.24–1.73)	1.78 (1.49–2.06)
Cancer**					
Sexual minority	9.2 (8.4–10.0)	7.9 (5.6–10.2)	9.2 (8.4–9.9)	9.1 (6.5–11.6)	9.7 (6.2–13.3)
Heterosexual	7.3 (7.2–7.4)	6.1 (5.8–6.5)	7.8 (7.6–7.9)	5.8 (5.3–6.4)	5.9 (5.4–6.5)
aPR	1.26 (1.15–1.37)	1.29 (0.90–1.67)	1.18 (1.08–1.28)	1.56 (1.10–2.02)	1.64 (1.02–2.26)
**Heart disease^††^**					
Sexual minority	8.0 (7.3–8.9)	8.8 (6.2–11.4)	7.3 (6.6–8.0)	10.9 (7.8–14.1)	9.8 (6.6–13.0)
Heterosexual	6.8 (6.6–6.9)	7.0 (6.6–7.4)	6.7 (6.6–6.8)	7.0 (6.5–7.5)	6.7 (6.1–7.3)
aPR	1.19 (1.08–1.30)	1.26 (0.88–1.64)	1.09 (0.98–1.19)	1.56 (1.10–2.03)	1.46 (0.97–1.95)
**COPD**					
Sexual minority	10.3 (9.5–11.1)	10.2 (7.8–12.7)	10.1 (9.4–11.1)	9.2 (7.1–11.3)	10.3 (7.3–13.3)
Heterosexual	6.9 (6.8–7.0)	7.1 (6.7–7.6)	7.3 (7.2–7.5)	5.9 (5.4–6.3)	4.8 (4.3–5.3)
aPR	1.49 (1.37–1.61)	1.44 (1.09–1.78)	1.40 (1.28–1.52)	1.45 (1.14–1.76)	2.15 (1.49–2.81)
**Diabetes**					
Sexual minority	12.5 (11.6–13.4)	18.5 (15.3–21.7)	11.0 (10.1–11.9)	17.4 (13.1–21.6)	14.6 (11.4–17.7)
Heterosexual	11.6 (11.4–11.7)	17.1 (16.6–17.7)	9.8 (9.6–9.9)	13.7 (12.9–14.4)	16.1 (15.3–16.8)
aPR	1.08 (1.00–1.16)	1.08 (0.89–1.27)	1.12 (1.03–1.22)	1.27 (0.95–1.59)	0.91 (0.71–1.11)
**Hypertension^§§^**					
Sexual minority	35.7 (34.2–37.1)	45.4 (41.5–49.4)	34.9 (33.2–36.5)	35.9 (30.1–41.1)	32.3 (27.4–37.2)
Heterosexual	33.6 (33.3–33.9)	45.2 (44.3–46.1)	32.1 (31.8–32.4)	31.0 (29.7–32.2)	32.1 (31.1–33.1)
aPR	1.06 (1.02–1.11)	1.01 (0.92–1.09)	1.09 (1.05–1.14)	1.16 (0.98–1.33)	1.01 (0.85–1.16)
**Kidney disease**					
Sexual minority	4.7 (4.0–5.4)	7.2 (4.2–10.2)	4.2 (3.6–4.8)	4.5 (3.0–5.9)	5.8 (2.7–8.8)
Heterosexual	3.2 (3.1–3.3)	4.2 (3.8–4.5)	2.9 (2.8–3.0)	3.6 (3.0–4.1)	3.7 (3.3–4.1)
aPR	1.47 (1.25–1.69)	1.73 (0.99–2.46)	1.42 (1.22–1.63)	1.25 (0.80–1.70)	1.55 (0.71–2.39)
**Obesity (BMI≥30 kg/m^2^)**					
Sexual minority	34.1 (32.9–35.3)	41.4 (37.6–45.1)	33.6 (32.3–35.0)	26.2 (22.8–29.7)	35.4 (31.5–39.3)
Heterosexual	31.9 (31.6–32.1)	41.0 (40.2–41.8)	30.5 (30.2–30.7)	22.1 (21.1–23.0)	35.4 (34.4–36.5)
aPR	1.07 (1.03–1.11)	1.01 (0.92–1.10)	1.10 (1.06–1.15)	1.19 (1.03–1.35)	1.00 (0.89–1.11)
**Smoking, current**					
Sexual minority	22.1 (21.1–23.1)	22.4 (19.4–25.4)	22.9 (21.7–24.0)	19.1 (16.2–22.0)	19.1 (16.0–22.2)
Heterosexual	15.5 (15.3–15.7)	16.9 (16.3–17.5)	16.5 (16.3–16.7)	12.8 (12.2–13.5)	11.5 (10.8–12.1)
aPR	1.43 (1.36–1.50)	1.32 (1.14–1.51)	1.39 (1.31–1.46)	1.49 (1.25–1.73)	1.67 (1.38–1.95)
**Stroke**					
Sexual minority	4.7 (4.1–5.4)	7.5 (5.1–9.9)	4.0 (3.4–4.5)	5.7 (3.2–8.2)	6.2 (3.5–8.9)
Heterosexual	3.4 (3.4–3.5)	5.5 (5.2–5.9)	3.2 (3.1–3.3)	3.6 (3.2–4.0)	3.0 (2.6–3.4)
aPR	1.37 (1.19–1.56)	1.36 (0.91–1.81)	1.24 (1.05–1.43)	1.59 (0.88–2.30)	2.08 (1.13–3.00)

**Abbreviations:** BMI = body mass index; CI = confidence interval; COPD = chronic obstructive pulmonary disease.

* Adjusted for age, sex (female or male), and Behavioral Risk Factor Surveillance System survey year. Adjusted prevalence ratios with 95% CIs that exclude 1 are statistically significant.

**^†^** Includes conditions with strong or mixed evidence of associations with COVID-19–associated adverse outcomes.

**^§^** Includes persons who identified as gay, lesbian or gay, or bisexual. The analysis excludes those who responded to the sexual orientation question with “something else,” “other,” or “don’t know” or who refused (3.4% of respondents).

^¶^ Unweighted number of respondents.

** Lifetime history of cancer, except nonmelanoma skin cancer.

**^††^** Includes heart attack/myocardial infarction, coronary heart disease, or angina.

**^§§^** 2017 and 2019 Behavioral Risk Factor Surveillance Systems only.

## Discussion

This analysis found that several underlying health conditions that increase or might increase the risk for more severe COVID-19–related illness were more prevalent among sexual minority persons than heterosexual persons, both within the overall population and within specific racial/ethnic groups. Non-Hispanic Black and Hispanic populations have been disproportionately affected by the COVID-19 pandemic in the United States, and the increased prevalence of certain risk factors among sexual minority members of these racial/ethnic minority populations is of particular concern. Because of their sexual orientation, sexual minority persons experience stigmatization and discrimination (*1*) that can increase vulnerabilities to illness and limit the means to achieving optimal health and well-being through meaningful work and economic security, routine and critical health care, and relationships in which sexual orientation and gender identity can be openly expressed (*5*). Persons who are members of both sexual minority and racial/ethnic minority groups might therefore experience a convergence of distinct social, economic, and environmental disadvantages that increase chronic disease disparities and the risk for adverse COVID-19–related outcomes.

In November 2020, CDC conducted a series of group listening sessions with representatives of advocacy and health care organizations serving sexual and gender minority communities across the United States to gather information on the effect of the pandemic on their constituents and patient populations. A major concern expressed in these sessions was that information about sexual orientation and gender identity is not standard in COVID-19 data collection systems. Privacy issues around sexual orientation and concerns about nonresponse or refusals to answer such questions have often been used as justification for not including these elements in public health surveillance and patient record systems (*6*); however, regarding public health surveillance systems, CDC surveys such as BRFSS, the National Health Interview Survey, and the National Survey on Family Growth have demonstrated the feasibility of collecting sexual orientation data from the civilian, noninstitutionalized population on an ongoing basis (*2*). Several months into the COVID-19 pandemic, several states and local jurisdictions responded to demands from advocacy organizations to begin collecting these data. For example, in July 2020, California Health and Human Services announced emergency regulations that required local health departments and service providers to collect and report voluntary data on sexual orientation and gender identity to better understand the effect of COVID-19 in these population subgroups. Illinois has included a COVID-19 module in its 2020 BRFSS that also includes questions on sexual orientation and gender identity (*7*). Pennsylvania, the District of Columbia, and several other jurisdictions are taking steps toward including sexual orientation and gender identity information in COVID-19–related data collection; however, these data are not yet available (*6*).

The findings in this report are subject to at least six limitations. First, all conditions are self-reported, and all but three (asthma, obesity, and smoking) refer to lifetime instead of current prevalence. Second, although the 3-year data set included as many as 31 states in 2019, the data are not nationally representative. Third, although BRFSS variables used in this analysis are general measures of the list of underlying health conditions identified by CDC as COVID-19 risk factors (*3*), they do not always reflect the clinical specificity of the condition list; for example, the diabetes question does not distinguish between type 1 and type 2 diabetes, and the heart disease variable includes conditions that might not affect COVID-19 outcomes (*4*). Fourth, several important underlying health conditions, such as sickle cell disease, have no corresponding variable in BRFSS. Fifth, although BRFSS includes a question on gender identity, the number of respondents identifying as transgender or nonbinary was too small for reliable estimates compared with the majority cisgender population. Finally, the large number of respondents in the aggregated non-Hispanic other race/ethnicity category could potentially obscure disparities between sexual minority and heterosexual populations within these smaller communities.

Despite the numerous studies among racial/ethnic minority groups and the increasing number of studies among sexual minority groups, examinations of health outcomes by combinations of sexual orientation and race/ethnicity remain relatively rare. Attention to potentially larger disparities at the intersections of sexual orientation and race/ethnicity is critical to ensuring health equity for all, including subpopulations whose circumstances often remain uncaptured despite acknowledgments of their distinct importance and needs. Because of longstanding social inequities and higher prevalences of several underlying health conditions, sexual minority populations might be vulnerable to COVID-19 acquisition and associated severe outcomes, and this vulnerability might be magnified when coupled with other demographic characteristics such as race/ethnicity (*8*). However, because data on sexual orientation are not collected in existing COVID-19 data systems, the effect of COVID-19 on sexual minority populations is unknown. This data gap underscores the need to extend COVID-19 surveillance and other studies to include measures of sexual orientation and gender identity. This recommendation is consistent with the emphasis on “key equity indicators” in the January 2021 Executive Order on Ensuring a Data-Driven Response to COVID-19 and Future High-Consequence Public Health Threats (*9*). Expanding sexual orientation and gender identity data collection to surveillance systems with shorter lags in data reporting could support more equitable representation of sexual and gender minority populations in public health data systems to facilitate improved decision-making during and after the pandemic.

**Summary**

**What is already known about this topic?**

Risks for COVID-19 acquisition and severe associated illness vary by characteristics, including race/ethnicity, age, and urban/rural residence. U.S. COVID-19 surveillance systems lack information on sexual orientation, hampering examination of COVID-19–associated disparities among sexual minority adults.

**What is added by this report?**

Sexual minority persons in the United States have higher self-reported prevalences of several underlying health conditions associated with severe outcomes from COVID-19 than do heterosexual persons, both in the overall population and among racial/ethnic minority groups.

**What are the implications for public health practice?**

Inclusion of sexual orientation and gender identity data in COVID-19 surveillance and other data collections could improve knowledge about disparities in infections and adverse outcomes among sexual and gender minority populations, overall and by race/ethnicity.

## Footnotes

* BRFSS collects information on demographics and health, including underlying conditions, use of preventive services, health care access, and health-related behavioral risk factors for the 50 states, District of Columbia, three U.S. territories (American Samoa, Guam, and U.S. Virgin Islands), and two freely associated states (Federated States of Micronesia and Palau). https://www.cdc.gov/brfss/index.html

† Health conditions were elicited by the question “Has a doctor, nurse, or other health practitioner ever told you that you have…” followed by a set of conditions, including those used in this analysis: a heart attack, also called a myocardial infarction; angina or coronary heart disease; stroke; asthma (with positive responses followed by “Do you still have asthma?”); any other type of cancer (other than skin cancer); chronic obstructive pulmonary disease, emphysema, or chronic bronchitis; kidney disease (not including kidney stones, bladder infection, or incontinence); or diabetes (followed by questions allowing separation of gestational diabetes, prediabetes, and borderline diabetes). Hypertension was assessed separately but with the same question format. Possible responses to these questions were “yes,” “no,” “don’t know/not sure,” or refused. Because coronary heart disease is the most common cause of heart attack/myocardial infarction, respondents answering affirmatively to questions about a heart attack/myocardial infarction, angina, or coronary heart disease were counted as having heart disease.

§ Jurisdictions with a sexual orientation question in 2017 were California, Connecticut, Delaware, Florida, Georgia, Hawaii, Illinois, Indiana, Iowa, Louisiana, Massachusetts, Minnesota, Mississippi, Montana, Nevada, New York, North Carolina, Ohio, Oklahoma, Pennsylvania, Rhode Island, South Carolina, Texas, Vermont, Virginia, Washington, Wisconsin, and Guam. Jurisdictions with a sexual orientation question in 2018 were Connecticut, Delaware, Florida, Hawaii, Idaho, Illinois, Kansas, Louisiana, Maryland, Minnesota, Mississippi, Missouri, Montana, Nevada, New York, North Carolina, Ohio, Oklahoma, Pennsylvania, Rhode Island, South Carolina, Tennessee, Texas, Vermont, Virginia, Washington, West Virginia, Wisconsin, and Guam. Jurisdictions with a sexual orientation question in 2019 were Alaska, Arizona, Colorado, Connecticut, Delaware, Florida, Georgia, Hawaii, Idaho, Indiana, Kansas, Louisiana, Maryland, Minnesota, Mississippi, Montana, New York, North Carolina, Ohio, Oklahoma, Rhode Island, South Carolina, Tennessee, Texas, Utah, Vermont, Virginia, Washington, West Virginia, Wisconsin, and Guam.

¶ In 2017, the BRFSS question on sexual orientation was “Do you consider yourself to be: straight, lesbian or gay, or bisexual?” The interviewer recorded responses of “other,” “don’t know/not sure,” and refusals. In 2018 and 2019, the BRFSS question on sexual orientation was “Which of the following best represents how you think of yourself?” Response options for men were “gay”; “straight, that is, not gay”; “bisexual”; and “something else.” Response options for women were “lesbian or gay”; “straight, that is, not gay”; “bisexual”; and “something else.” The interviewer recorded “don’t know” responses and refusals. The analysis excludes 3.4% of respondents who responded “something else,” “other,” or “don’t know” to the sexual orientation question or who refused to respond.

** The differences between the CDC list of conditions that place adults at increased risk for severe illness from COVID-19 and the BRFSS variables are the following, respectively: current cancer versus lifetime history of (ever) cancer; current chronic kidney disease versus ever kidney disease; current chronic obstructive pulmonary disease versus ever chronic obstructive pulmonary disease; current heart condition versus ever coronary heart disease, angina, or heart attack/myocardial infarction; obesity and severe obesity versus obesity; and current type 2 diabetes mellitus versus ever diabetes. BRFSS does not include a variable on sickle cell disease, which is one of the conditions on the CDC “at increased risk” list. The differences between the CDC list and BRFSS variables are the following, respectively: moderate to severe asthma versus asthma; current cerebrovascular disease versus ever stroke; current hypertension versus ever hypertension; and current type 1 diabetes mellitus versus ever diabetes.

## References

[1] Logie C. The case for the World Health Organization’s Commission on the Social Determinants of Health to address sexual orientation. Am J Public Health 2012;102:1243–6. 10.2105/AJPH.2011.300599 22594723 PMC3478032

[2] US Department of Health and Human Services. Healthy people 2020: lesbian, gay, bisexual, and transgender health. Washington, DC: US Department of Health and Human Services; 2020. https://www.healthypeople.gov/2020/topics-objectives/topic/lesbian-gay-bisexual-and-transgender-health

[3] CDC. COVID-19: people with certain medical conditions. Atlanta, GA: US Department of Health and Human Services, CDC; 2020. https://www.cdc.gov/coronavirus/2019-ncov/need-extra-precautions/people-with-medical-conditions.html

[4] Silver SR, Li J, Boal WL, Shockey TL, Groenewold MR. Prevalence of underlying medical conditions among selected essential critical infrastructure workers—Behavioral Risk Factor Surveillance System, 31 states, 2017–2018. MMWR Morb Mortal Wkly Rep 2020;69:1244–9. 10.15585/mmwr.mm6936a3 32914769 PMC7499835

[5] Flentje A, Heck NC, Brennan JM, Meyer IH. The relationship between minority stress and biological outcomes: a systematic review. J Behav Med 2020;43:673–94. 10.1007/s10865-019-00120-6 31863268 PMC7430236

[6] Krause KD. Implications of the COVID-19 pandemic on LGBTQ communities. J Public Health Manag Pract 2021;27(Suppl 1):S69–71. 10.1097/PHH.0000000000001273 33239566

[7] Cahill S, Grasso C, Keuroghlian A, Sciortino C, Mayer K. Sexual and gender minority health in the COVID-19 pandemic: why data collection and combatting discrimination matter now more than ever. Am J Public Health 2020;110:1360–1. 10.2105/AJPH.2020.305829 32783729 PMC7427229

[8] Gray DM 2nd, Anyane-Yeboa A, Balzora S, Issaka RB, May FP. COVID-19 and the other pandemic: populations made vulnerable by systemic inequity. Nat Rev Gastroenterol Hepatol 2020;17:520–2. 10.1038/s41575-020-0330-8 32541960 PMC7294516

[9] Executive Office of the President. Ensuring a data-driven response to COVID-19 and future high-consequence public health threats. Fed Regist 2021 Jan 26;86(15):7189–91. https://www.federalregister.gov/documents/2021/01/26/2021-01849/ensuring-a-data-driven-response-to-covid-19-and-future-high-consequence-public-health-threats

